# Could Seals Prevent Cod Recovery in the Baltic Sea?

**DOI:** 10.1371/journal.pone.0018998

**Published:** 2011-05-09

**Authors:** Brian R. MacKenzie, Margit Eero, Henn Ojaveer

**Affiliations:** 1 National Institute for Aquatic Resources, Technical University of Denmark (DTU-Aqua), Charlottenlund, Denmark; 2 Department of Biology, Center for Macroecology, Evolution and Climate, University of Copenhagen, Copenhagen, Denmark; 3 Estonian Marine Institute, University of Tartu, Pärnu, Estonia; University of Glamorgan, United Kingdom

## Abstract

Fish populations are increasingly affected by multiple human and natural impacts including exploitation, eutrophication, habitat alteration and climate change. As a result many collapsed populations may have to recover in ecosystems whose structure and functioning differ from those in which they were formerly productive and supported sustainable fisheries. Here we investigate how a cod (*Gadus morhua*) population in the Baltic Sea whose biomass was reduced due to a combination of high exploitation and deteriorating environmental conditions might recover and develop in the 21st century in an ecosystem that likely will change due to both the already started recovery of a cod predator, the grey seal *Halichoerus grypus*, and projected climate impacts. Simulation modelling, assuming increased seal predation, fishing levels consistent with management plan targets and stable salinity, shows that the cod population could reach high levels well above the long-term average. Scenarios with similar seal and fishing levels but with 15% lower salinity suggest that the Baltic will still be able to support a cod population which can sustain a fishery, but biomass and yields will be lower. At present knowledge of cod and seal interactions, seal predation was found to have much lower impact on cod recovery, compared to the effects of exploitation and salinity. These results suggest that dual management objectives (recovery of both seal and cod populations) are realistic but success in achieving these goals will also depend on how climate change affects cod recruitment.

## Introduction

Humans have been impacting marine ecosystems for 1000s of years and have caused a number of populations of exploited marine animals to decline to low levels or become extirpated [1], [2]. Rebuilding populations from low levels requires low or zero exploitation rates [3]; however even such strategies may not always guarantee a recovery of collapsed fish populations [4], [5]. This is partly due to changes in population biology (e. g., Allee effects, changes in age or size structure), in the foodweb (predator-prey interactions, competition) or abiotic conditions in the ecosystem (e. g., temperature, oxygen conditions; habitat alteration) which impair survival [6]–[8]. As a result, the ecosystem into which collapsed populations are expected to re-occupy may no longer have the same properties conducive for survival and productivity as when the populations were larger. In these circumstances, reduced exploitation by itself may be a necessary but insufficient management measure to ensure population recovery [9].

**Figure 1:**
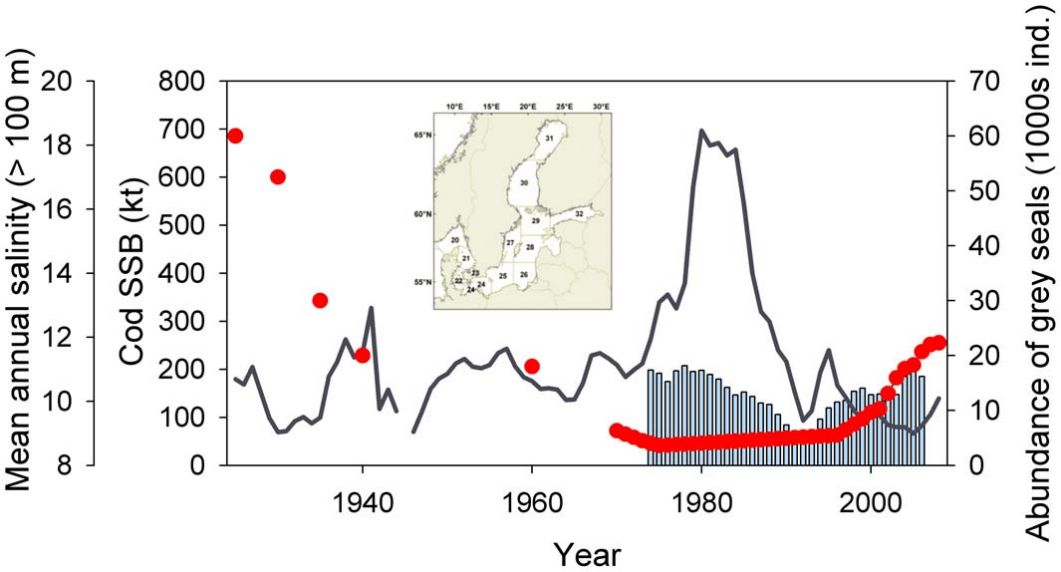
Temporal development of cod spawner biomass [**52**] (line), salinity in the deep layer of the Baltic Sea (>100 m in Landsort Deep; bars [**31**]) and grey seal abundance in the Baltic Sea (red circles )[**42**]. Inset shows map of the Baltic Sea with ICES Subdivisions indicated.

This situation could apply to cod, *Gadus morhua*, in the eastern Baltic Sea (Subdivisions 25–32 of the International Council for the Exploration of the Sea, ICES). The biomass of this population has been at or below its long-term mean since the mid 1980s, reached record low level in the early 2000s, and has started to increase in recent few years (Figure 1) [10]. Management strategies which can promote cod recovery and long-term sustainable fisheries have been and are being discussed in international working groups and management agencies [11]–[14], and evaluated via simulation modelling [15]–[18]. In the short-medium term (i.e., 5–10 years), the main strategy is to reduce exploitation, and improve compliance with the regulations by the fishing industry. However at longer time scales (multi-decadal), other ecosystem issues that potentially will affect cod biomass may have to be addressed and incorporated in long-term management strategies and policies.

One of these issues is a recovery of grey seals that is part of the Baltic Sea Action Plan of the Baltic Marine Environment Protection Commission (HELCOM BSAP) to achieve good ecological status of the Baltic Sea [19]. Further, the recovery of cod biomass to the level where it can provide maximum sustainable yields is part of management objectives for the Baltic Sea [12], [19], [20]. Seals are however predators of cod [21] and have historically had a substantial impact on cod biomass [22], [23]. Seals are believed responsible for delayed recovery of some collapsed cod populations elsewhere [7], [24], [25], and may even be contributing to the predicted extirpation within the next 4 decades of one of those population [7]. Given these interactions and the generality of top-down controls in marine foodwebs [26], the two management objectives concerning seals and cod in the BSAP may be contradictory [22], [27] and difficult to achieve simultaneously.

A second issue which will affect the Baltic Sea in future is projected climate change, which will lead to warmer temperatures, potentially lower salinity [28] and will interact with ongoing eutrophication problems [19], [28]. The main and most direct negative effect of climate change on the cod population, and more generally on the marine species in this species-poor brackish system [29], could be the reduction in salinity [28], which would negatively impact reproduction success (especially the survival of eggs and larvae) [30], [31].

Conditions for cod production in the Baltic will therefore likely become worse in the coming decades both due to increased seal predation and forecasted climate change. However the magnitudes of these impacts in relation to each other and in relation to exploitation are presently unknown, as is the timing of when these impacts might occur. These forcings potentially have important consequences for the long-term biomass of cod in the Baltic Sea and consequently for the achievement of key ecosystem management objectives [19], including new policies being developed in the EU related to ecosystem-based management, Good Environmental Status, and Maximum Sustainable Yields [20], [32]. We have therefore investigated the dynamics of cod under possible future scenarios using simulation modelling. We wished to quantify the importance of seal predation on cod recovery and population development relative to cod exploitation and some preliminary forecasts of how climate change might affect cod reproduction and biomass.

## Methods

### General characteristics of simulation model

The biomass of cod in the Baltic Sea during the 21^st^ century was simulated using an age-structured stochastic analytical population model [33]–[35] which includes an environmentally-dependent stock-recruitment model and age-specific predation mortality due to seals (details below). Changes in numbers of cod from one year to the next were represented by standard stock numbers equations which form the basis for standard fish stock assessments [36]:
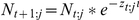
where N’s are numbers of individuals of age *j* and z = total mortality of age *j* (due to age-specific exploitation, F*_j_*, and natural mortality, M*_j_*; z = F+M).

The numbers of fish removed by fishing are represented by:
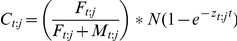
where C = catch in numbers of fish aged *j* [36].

The population development is projected forward by annually estimating the numbers of survivors from a previous year, The production of offspring (recruitment; i. e., the number of cod which survive to age 2) in each year included in the population model was estimated from an adult biomass-recruitment model (described below). The population development was simulated for the years 2009–2089.

Input data for initial stock numbers at age, individual weights, maturity, and natural mortality (in addition to seal predation) for age groups 2–8+ were obtained from the stock assessments conducted by the International Council for the Exploration of the Sea [37]. For initial numbers–at-age 2, we assumed a coefficient of variation (CV) of 0.40 [37]; CV of older ages was assumed to be 0.16 (age 3), 0.13 (ages 4–6) and 0.15 (ages 7+) [37]. Numbers-at-age were then estimated from a random lognormal distribution based on observed numbers-at-age and their variability. Weight at age, maturation and natural mortality due to other reasons than seal predation were assumed constant in projections because functional relationships describing their variability are unknown [37].

Adult biomass (spawners) was estimated annually from numbers of fish, their probability of being mature and their weights. Each projection for a given combination of inputs and forcings was repeated 200 times to accommodate uncertainty in initial population numbers-at-age and recruitment. Outputs are probabilistic estimates of numbers-at-age, biomass and fishery yields. The frequency distribution of spawner biomass in 2089 from the 200 runs was used to estimate the probability that final simulated spawner biomass would exceed the long-term mean during 1925–2008 [23]. Maximum spawner biomass was restricted to the historically observed maximum during 1925–2008 (700,000 t; Figure 1).

**Cod recruitment model.** Several previous studies have shown that cod recruitment in the Baltic Sea is functionally related to both spawner biomass and environmental variability (e. g., [16], [30], [31]). The main environmental factors that affect Baltic cod recruitment are salinity and oxygen conditions, both of which are expected to change during the 21^st^ century due to changes in climate and nutrient loading to the Baltic Sea [28], [30]. However the magnitude and timing of such changes are still uncertain due to incomplete knowledge of the processes which affect both the physical oceanography and the biogeochemical cycling of the Baltic Sea, and because different climate-hydrographic models give different results [28], [38]. The stock-recruitment-environment model used in this study includes salinity as a proxy for hydrographic conditions. Such a model has recently been shown to explain significant amount of past recruitment variation [31]. The model used here is similar to that employed by [31], except that our model estimates numbers of 2-year old cod, instead of 0-group (<1 year old) cod, and includes 2 additional recent years of data (i. e., yearclasses born in 1974–2006). The model implemented and its associated statistics are given below:
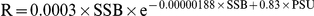
where R = recruitment (1000s of 2-year old cod), SSB = spawner biomass, PSU = practical salinity unit (R^2^_adj._ = 0.64; P<0.0001; SE_est._ = 118794).

Predicted recruitment was calculated based on a log-gaussian distribution with variance of ln residuals, 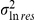. Salinity data included in the model were annual averages of salinity for depths>100 m at the Landsort Deep monitoring site and were compiled from data held at the Finnish Environment Institute, Marine Research Centre and Swedish Meteorological and Hydrological Institute (database SHARK) [31]. Salinity at this site is used in this study as an indicator of the complex processes through which salinity and oxygen concentration affect cod reproduction [39]. Salinity is a useful indicator for the purpose of this study because forecasts of developments of this variable are available.

In contrast, detailed representations of how oxygen concentration in cod spawning areas could fluctuate under future climate change are still being developed and somewhat more uncertain [38]. In future, new climate change-driven coupled bio-physical models of the lower trophic levels of the foodweb could potentially provide better indicators of cod reproductive habitat quality which in turn could be used in new cod spawner biomass-recruitment-environment models.

Coupled climate-ocean models for the Baltic Sea predict a future decrease in salinity ranging from 0 to ca. 50% [28], [38]. Consequently, our simulations assumed that future salinity would either remain stable (i.e., equivalent to its long-term mean value, 10.2, during 1974–2006 but with random variability as defined below) or decrease. However a decrease by 50% would produce salinities below those observed in available time series [31], and if used in stock-recruitment-environment model could introduce additional uncertainty to the results. We therefore restricted the decline in salinity to the minimum observed value during 1974–2006 (8.7, or 15%) and assumed that the average decline rate between the present and future (2009 and 2089) was 0.019 (i. e., 1.5/81 years).

We assumed that the variability in future salinity was similar to that in the past, which includes both autocorrelated and random processes [8]. Hence future salinity was estimated by adding past variation to the expected trend [8] according to 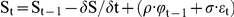
where S = annual salinity, δS/δt = mean rate of change of salinity (0.019), ρ = first order (lag 1) autocorrelation (0.66; [8]), *ϕ*_t−1_ = autocorrelated component of salinity variation (initial value assumed = 0), σ = standard deviation of observed time series of salinity during 1974–2006, ε_t_ = random number (mean = 0; range = −1−1). If predicted S_t_<8.7, then S_t_ was assumed 8.7.

Forecasted salinities were used as inputs to the Ricker stock-recruitment-environment model to estimate recruitment.

### Estimation of seal population development and consumption of cod in the 21^st^ century

There are three species of seals living in the Baltic Sea (grey seals *Halichoerus grypus*, ringed *Phoca hispida*, and harbour seals *Phoca vitulina*). Here we consider only the grey seals because this species is presently most abundant, has increased 8%/year in recent years [40] and is distributed most southerly in the Baltic and therefore most likely to overlap with and encounter cod. The developmental trajectory of the grey seal population in the 21^st^ century is unknown. We estimated its trajectory using a simple logistic model of population growth applied to recent population growth rate and historical abundance data. The model was fitted to observed data from 2000–2008 [41] and a maximum final population estimate in 2089 that we assumed to be at the level corresponding to abundances observed in the early 1900s (i. e., 90,000 individuals [42]). The fitted relationship was y = 90,000/{1+(x/2016)^−248^}.

The average food requirement of grey seals in the Baltic has been estimated to be 3.2 kg/seal/day [43]. According to the historical observations, the contribution of cod to the seal diet has varied over time, in line with changed cod abundance. Cod (mainly young individuals) comprised less than 5% of grey seal diets in the early 2000s [44] but ∼20% in the 1960s–1970s [45], similar to what has been observed in other regions ([46]). The abundance of cod in the Baltic was different in the two time periods [47] and we hypothesize that the higher proportion of cod in seal diet was due partly to the higher abundance of young cod in the former period (291 vs. 120 million). Hence, we assumed in our simulations that cod proportion in seal diet increases linearly with cod recruit abundance up to a maximum of 30% of cod in seal diets.

The typical length of fish consumed by grey seals in the Baltic is 10–25 cm [44]. In the North Sea, most fish consumed by grey seals are <30 cm [46], and grey seals in Atlantic Canada also consume mainly juvenile cod [25], [48]. Cod of this size range (10–30 cm) are 1–2 years old and weigh ca. 0.2 kg in the Baltic Sea. Seal dietary composition is usually derived from remains of hard parts (bones, otoliths) in seal stomachs or feces; however, grey seals may also prey on larger sizes of cod by consuming only soft portions of cod carcasses (“belly-biting”; [48]). The incidence of this predation behaviour has however not been quantified for the Baltic and therefore is not included in our simulations.

The biomass of cod consumed by seals per day was calculated multiplying the daily food requirement of seals with the number of seals and the proportion of cod in the diet, which was scaled to annual values and converted to numbers of cod using assumed weight of captured cod (0.2 kg). Seal predation on cod was implemented in the cod population projection model assuming that the predation occurred at and before the start of each year for age 2 cod. Hence predicted annual recruitment from the recruitment model was reduced by seal predation before applying fishing and other natural mortality to remaining survivors throughout the rest of the year.

### Scenarios

We conducted simulations of scenarios which investigated how the cod population would react to different combinations of seal predation mortality, climate change (salinity decrease), and exploitation.

The exploitation levels applied in the scenarios included the target level in the cod management plan (i. e., F_mp_ = 0.3) for the eastern Baltic Sea [12] and 2- and 3-fold higher levels (i. e., F = 0.3, 0.6 and 0.9) for the main age groups in the fishery (4–7). Relative fishing mortality of younger ages was assumed to be similar to the mean level observed in1966–2009) [37]. Scenarios are summarized in Table 1.

**Table 1:** Scenarios employed to simulate cod population dynamics in the eastern Baltic Sea (ICES Subdivisions 25–32) during the 21^st^ century for different combinations of exploitation, salinity (as a consequence of expected climate change) and seal predation.

Scenario	Fishing mortality	Seal predation	Salinity
1	0.3	Low	Long-term mean
2	0.6	Low	Long-term mean
3	0.9	Low	Long-term mean
4	0.3	Low	Decrease 0.019/yr
5	0.6	Low	Decrease 0.019/yr
6	0.9	Low	Decrease 0.019/yr
7	0.3	Increasing	Long-term mean
8	0.6	Increasing	Long-term mean
9	0.9	Increasing	Long-term mean
10	0.3	Increasing	Decrease 0.019/yr
11	0.6	Increasing	Decrease 0.019/yr
12	0.9	Increasing	Decrease 0.019/yr

The categories “low” for the seal predation rate refer to the present level of seal predation which is part of the overall natural mortality [37]; i. e., no additional seal predation mortality was imposed on cod for these simulations. The seal predation category “increasing” refers to the increasing predation on cod from seals that occurs as the seal population increases during the 21^st^ century to its historical abundance level. This additional predation is added to other sources of natural mortality for cod. The long-term mean for salinity is for the period 1974–2006.

The relative effects of increased seal predation, reduced salinity and increased fishing mortality on cod spawner biomass in the end of the simulation period were calculated by comparing spawner biomass from respective scenarios with the reference scenario, i.e. stable seal predation and salinity and fishing mortality corresponding to F_mp._

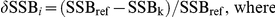
SSB_ref_ = spawner biomass under management plan exploitation with low seal predation and constant salinity ;

SSB_k_ = spawner biomass with realized change in one of the three forcings, i.e. either increased seal predation, salinity reduction or increased fishing.

The three δSSB*_i_* values were then scaled relative to 1 for comparison.

## Results

**Figure 2:**
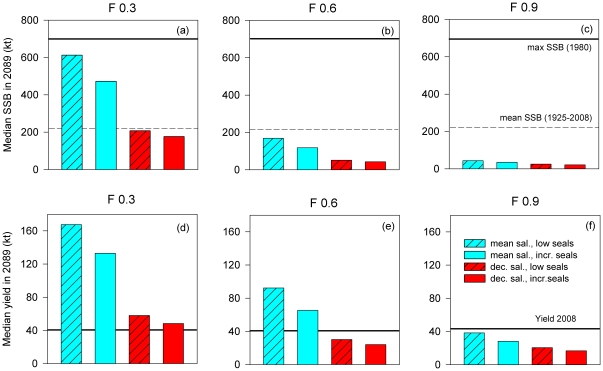
Simulated cod biomass and fishery yield in the 21^st^ century in the eastern Baltic Sea for different combinations of exploitation, climate change and seal predation. Panels a–c: Median projected cod spawner biomass in the eastern Baltic Sea (ICES Subdivisions 25–32) in 2089 estimated for different scenarios of exploitation level (F), seal predation and climate change (salinity decrease). The long-term (1925–2008) mean spawner biomass (220,000 t; solid horizontal line [37]) and the maximum historically observed spawner biomass since the 1920s (dashed horizontal line; [37], [52]) are shown for comparison. Panels d–f: Median projected yields of cod to the fishery in the Baltic Sea (ICES Subdivisions 25–32) in 2089 estimated for different scenarios of exploitation (F), seal predation and climate change (salinity decrease). Also shown for comparison is the yield in 2008 (solid horizontal line).

**Figure 3:**
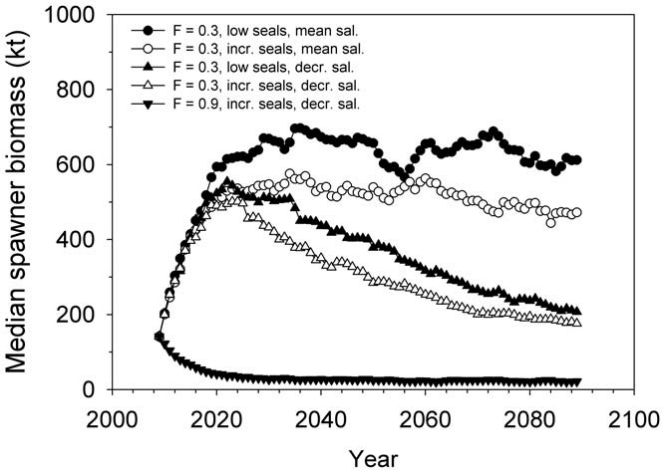
Temporal development of the projected median cod spawner biomass in the eastern Baltic Sea for different combinations of forcings (exploitation, seal predation, climate change induced salinity decline). See Methods and Table 1 for modelling details.

**Figure 4:**
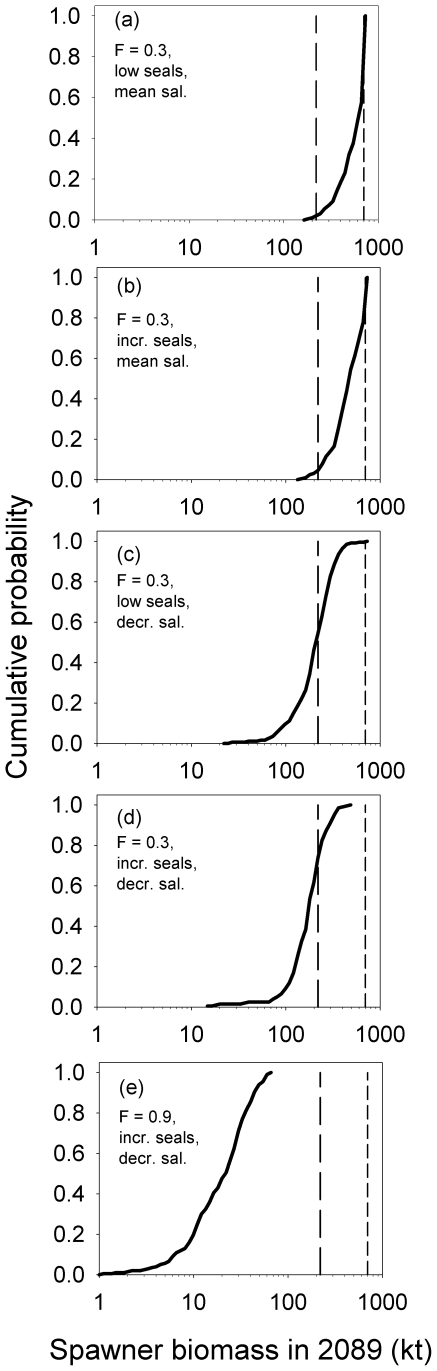
Cumulative probability of cod spawner biomass in the eastern Baltic Sea in 2089. Different panels show outputs for different combinations of forcings (exploitation, seal predation, climate change induced salinity decline) as derived from 200 stochastic simulations of an age-structured population model. The vertical long-dashed and short-dashed lines represent respectively the long-term mean and maximum spawner biomass during 1925–2008 [23]. See Methods and Table 1 for modelling details.

The simulations showed that if exploitation in the future was at the management plan target level (0.3) and seal predation and salinity remained as they have been during 1974–2006, cod spawner biomass would increase to ca. 600,000 t, i.e. ca. 3 fold higher than the long-term mean (Figure 2a, 3). Spawner biomass rises quickly before plateauing after 2020–2030 (Figure 3), and there is nearly 100% probability that the biomass at the end of the simulation period (2089) would exceed the long-term mean level; there is ca. 10% chance that the biomass would reach the long-term maximum (Figure 4a). Median yield in 2089 would be nearly ca. 160,000 t or ca. 4-fold higher than in 2008 (Figure 2b).

Under changed ecosystem conditions, spawner biomass and yields would be lower. If the seal predation increased, but salinity remained at past levels, then final spawner biomass and yield corresponding to exploitation at F_mp_ would be ca. 470,000 t and 130,000 t, respectively, i.e. ca. 25% lower compared to the scenario assuming no seal recovery (Figure 2b), but still more than double the long-term mean spawner biomass and yield in 2008. There is a 95% chance that the cod spawner biomass would exceed the long-term mean (Figure 4b).

All scenarios assuming a 15% reduction of salinity to historically-observed minimum levels indicate a further reduction in spawner biomass and yields compared to the long-term mean or 2008 levels, and much higher probabilities (≥50%) that spawner biomass would fall/remain below the long-term mean (Figure 3, 4 c–e). Spawner biomass would most likely be ca. 200,000 t after an initial rise under F_mp_ in absence of seals (Figure 2, 3); if seals are present then spawner biomass would be ca. 170,000 t (Figure 2, 3) and there is 75% probability that spawner biomass will be less than the long-term mean (Figure 4d), and thus well below the long-term maximum biomass. Yields corresponding to these two scenarios are also much lower (respectively 58,000 and 48,000 t; Figure 2) than if salinity remains unchanged.

Higher levels of exploitation (F = 0.6 or 0.9) in combination with seal predation and reduced salinity reduce the cod population to very low levels (22,000–43,000 t; Figure 2, 3, 4e). Such a population could only support a very low-yield fishery (expected annual yields ca. 16,000–24,000 t). A “worst-case” scenario assuming F = 0.9, increased seal predation and 15% reduction in salinity would lead to a population which would have very little chance of exceeding the long-term mean (Figure 4e). Such a population would decline immediately from its current level without any increase (Figure 3).

**Figure 5:**
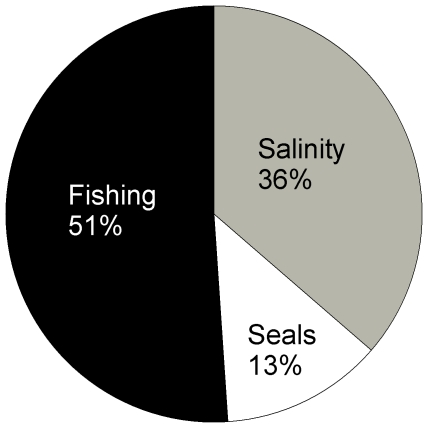
The relative effects of increased seal predation, a 15% decline in salinity and increased fishing up to 0.9 on projected cod spawner biomass in 2089. The fractions represent the relative contributions to the decline in biomass that is expected to occur relative to the biomass estimated assuming low seal predation, unchanged salinity and fishing mortality at 0.3.

Figure 5 shows the relative contributions of increased seal predation, reduced salinity and high fishing mortality to the reduction in cod spawner biomass from the level corresponding to the reference scenario, i.e. constant seal predation and salinity and fishing mortality at F_mp_. The relative effect of seal predation was only 13 percent, while reduction in salinity and increased fishing mortality to the level 0.9 contributed 36 and 51 percent to the biomass reduction, respectively.

## Discussion

### General

There are various projections available for the future development of the eastern Baltic cod population for the coming decades [8], [14], [16], [18], [27], [49], but we are not aware of any which have simultaneously considered the effects of recovery of seals in combination with exploitation and potential climate change. Our simulations thus provide new insight into the combined impacts of these three factors on a large predatory marine fish population living in a physiologically-stressed environment at the border of its species distribution area.

The projected development of the eastern Baltic cod population depends strongly on all three forcings considered. The status quo situation (modest exploitation, very low seal predation; mean salinity), if projected forward would (not surprisingly) give largest biomasses and yields, some of which would reach historical high levels. The initial rise in simulated biomass at F = 0.3 occurs because this level of F is lower than that which has occurred during most of the past 20–30 years and is allowing the population to rebuild after a period of low biomass [10]. However as described in the Introduction and Methods, this scenario is probably too optimistic for the entire 21^st^ century because of increasing abundance of seals and likely changes in abiotic conditions that affect cod reproduction. In particular, accounting for increased seal predation and climate change effects reduced expected biomasses and yields considerably, though with different magnitudes.

Perhaps the two most likely scenarios are those in which exploitation is maintained at (or close to) F_mp_, and the seal abundance increases as assumed here, for the two salinity scenarios. If salinity remains constant (as is forecast under some model simulations; [28]), then median spawner biomass and yields would be 470,000 t and 120,000 t respectively. If salinity decreases to historically-observed minimum levels, then spawner biomass and yields would fall to much lower levels and have lower probability of remaining above the long-term mean. As a result, an initial preliminary forecast for the future development of the cod population and fishery yield in a Baltic Sea containing historically abundant levels of seals suggests a spawner biomass range between 176,000–470,000 t and yields between 48,000–130,000 t.

### Seal-cod interactions

Our findings suggest that the impact of seals on the cod biomass is relatively small compared to either exploitation or the assumed salinity change. Consequently seals are not likely a major factor that will prevent cod recovery in this system. This conclusion is partly due to the presently still low abundance of seals, particularly in areas of the Baltic Sea where cod are present (i. e., spatial overlap of cod and seals is still relatively low) and because we have configured the cod share of seal diets to be proportional to cod abundance. As a result, the proportion of cod in seal diet declines when cod are rare, so the top-down control on cod from seals is weakened as cod abundance declines. This assumption of a declining share of prey in predator diets as prey become rare is consistent with some limited field observations of predator-prey interactions involving large marine animals (e. g., fish, seals) [50], [51].

The scenario outputs are consistent with historical knowledge of the Baltic Sea foodweb, including cod-seal interactions. Recent reconstruction of cod spawner biomass back to the early 20^th^ century [52] showed that cod spawner biomass in the 1920s–1930s when seals were much more abundant than at present (Figure 1) was at the same level (200,000 t) as that forecast in 2089 in the scenario involving constant salinity, low to moderate fishing and high seal predation. In the late 1500s-early 1600s, seals were also more abundant than now and cod exploitation was low [53], [54]. Archival tax records of cod fishing in the Baltic Sea during this period showed that cod was abundant in northern areas of the Baltic Sea [54], where the population expands at higher abundances, as observed in the 1980s. Cod were also commercially important in the southern Baltic in the early 1600s [54]. Hence there are historical precedents for the combination of abundant cod and seals in the Baltic Sea. Our calculations suggest that such a combination could occur again, if environmental conditions are satisfactory for cod reproduction and cod exploitation remains at low-modest levels.

We caution however that our results are based on incomplete knowledge of many interactions between species (e. g., predator-prey interactions between seals and cod), between species and their environment, and of the climate-hydrographic system itself. For example, the role of prey-switching on seal diets as potential prey species change in relative abundance and spatial distribution is unknown. The relationship between cod abundance and cod share in seal diets which was implemented here, although based on (limited) field data, is considered to be a pragmatic step in an otherwise complex and poorly documented ecological process. Functional responses of predators to prey abundances (e. g., types I, II, III responses [55]) have important implications for prey dynamics and sometimes also foodweb structure [56], [57], but are difficult to quantify and distinguish, especially for generalist highly mobile predators like seals, and in wild systems where neither predator nor prey abundances and distributions can be controlled by the investigator [57], [58]. This situation also applies to the case of the Baltic grey seals and cod prey; new knowledge of grey seal functional responses to prey species could therefore improve models of predator-prey interactions. For example, if seals have a type III functional response to cod abundance, then it is unlikely they would push the population to extinction; however this could be an outcome if their functional response was type II or I [56], [57].

Our simulations focus on only cod and seals, excluding other abundant fish species in the Baltic foodweb (e. g., sprat, herring), partly because of the prominence of cod and seals in local biodiversity and ecosystem management policies and partly because of their strong ecological roles within the upper part of the Baltic foodweb. Also, adding complexity to models can sometimes obscure otherwise clear results [59]. Cod is by far the most important fish predator of herring and sprat [60]–[62]. Consequently, an increase in cod abundance would not only result in more (cod) prey for seals, but would via cod predation [62], reduce the abundance of other prey (herring, sprat) for seals. Indeed, this mechanism might be a factor which contributes to the increase in share of cod within seal diets as cod become more abundant.

These interactions, as well as those involving predation by sprat and herring on cod eggs and larvae [63], [64], between herring-sprat-zooplankton [65], and of cod with benthic prey (e. g. *Saduria entomon* [60], [61]), could be investigated by expanding the species representation and climate forcing in some existing multi-species modelling approaches such as Multi-species Virtual Population Analysis [62], Ecopath [27], stochastic time-series methodologies [8], and Bayesian Multi-Species Functional Response models [57], [66].

Our results regarding the effect of increased seal abundance on cod biomass are line with an earlier investigation on cod-seal interactions in the Baltic represented by foodweb modelling via Ecopath, which have included all the main components in the Baltic food-web [27]. In these analyses cod biomass decreased by ca. 20–25% as seal abundance increased to early 1900 levels, which is similar to our results (Figure 2). Both our and Hansson et al (2007) results for the Baltic differ from a forecast of future cod biomass in a different temperate ecosystem (southern Gulf of St. Lawrence) where grey seals have been increasing in abundance. In that ecosystem, cod are expected to become extirpated within the next 40 years even if cod fishing is eliminated, partly due to an increase in natural mortality rates, which may be associated with the rise in seal abundance [7].

### Climate change impacts on cod

Our results suggest that climate change will have stronger impacts on the cod population than a recovering grey seal population (Figure 5). Even a modest reduction in salinity by 15% will likely mean that there will be 400,000 t less cod than if salinity fluctuated around the mean level observed during 1974–2006 (Figure 2; 4a, c). The impact of decreasing salinity on cod becomes most evident after the mid-2020s (Figure 3) because the population until then is still rebuilding from the previous period of high F prior to the late 2000s [10]. However the lower salinities already after 1–2 decades start to impact recruitment and biomass. Larger reductions in salinity would likely mean even more severe and earlier impacts on cod and lead to lower biomasses than those forecast here. For example, the cod population could become extinct [8] under some of the more severe salinity reductions (ca. 50%; [28]) seen in some climate-ocean model projections for the Baltic. The analyses and simulations conducted here have not considered a scenario in which salinity would increase, because such a scenario is presently considered largely unlikely [28], and we were focusing here on comparative impacts of potentially negative developments of different forcings in the Baltic Sea which could prevent cod recovery.

Our representation of how climate change affects cod is likely an oversimplification. In particular, it excludes a direct effect of climate change on oxygen concentrations in cod spawning areas and instead assumes that oxygen concentrations are only correlated with salinity variations. This assumption is likely true during much of the past, but might be violated in situations when the interval between major inflows of oxygenated saline water from the North Sea is long so that oxygen conditions in deep saline layers decline [67]. As a result, in some years salinity could be sufficient for cod reproduction but oxygen could be consumed and concentration become too low. Violation of this assumption contributes to the residual variability of our spawner biomass recruitment relationship which is carried forward into our stochastic simulations. As a result new cod recruitment models with better parameterisation of the salinity-oxygen conditions affecting recruitment could result in scenario forecasts with less uncertainty. Moreover, in future, biogeochemical models of the lower trophic levels of the Baltic foodweb [38], [68] may be able to produce estimates of how both salinity and oxygen concentrations might change in the Baltic Sea under future climate change. Such estimates could be used with stock-recruitment models to make alternative forecasts of the population development.

The present simulations should therefore be considered as a preliminary step towards more reliable understanding of interactions between cod, seals, climate change and the fishery. As various ecological processes are not included in our analyses (a situation typical to most ecological modelling exercises), the exact biomasses and time trends are associated with uncertainties. However, the analyses are considered to provide useful indications on relative impacts of the three forcings, i.e. climate change, exploitation and seal predation on cod recovery in the eastern Baltic Sea.

Our results do indicate that recovery success of the eastern Baltic cod will depend less on seal predation than it will on maintenance of low exploitation and the severity of future climate change. and suggest that the two management objectives regarding recovery of seals and cod within the HELCOM Baltic Sea Action Plan could be met. Moreover, even with a 15% reduction in salinity a commercially viable cod population should still be present, but with a substantially reduced fishery in accordance with lower productivity. We note however that continued eutrophication [19], [27], [28] and warming [28] of the Baltic Sea could reduce cod productivity even further than we have estimated via effects on oxygen concentrations in cod spawning areas [28], [53]. Although our calculations exclude explicit incorporation of these effects, they indicate that managing Baltic cod fisheries for sustainable yields will require an integrated approach which incorporates ecosystem components such as predator-prey interactions, climate-hydrographic forcing and eutrophication [13], [18], [22], [27], [69]. Implementing low exploitation will be a necessary condition but perhaps not sufficient to ensure high productivity and sustainable yields for this population.

### Conclusions

Increased seal abundances alone will not likely prevent cod recovery in the eastern Baltic Sea. However, recovery of the eastern Baltic cod to sustainable levels in the 21^st^ century will depend on low exploitation and the severity of future climate change (and its synergistic impacts with eutrophication). These findings demonstrate the importance of adjusting exploitation rates and recovery expectations to changing ecosystem conditions and more generally of adapting an ecosystem approach to fisheries management. These results could contribute to new Maximum Sustainable Yield (MSY)-based fishery management and Good Environmental Status strategies for the cod population and ecosystem of the Baltic Sea [20], [70], [71] which are part of new EU policy initiatives [32], [72].
